# Oral Health Status, Oral Health Behaviors, and Oral Health Care Utilization among Persons with Disabilities in Saudi Arabia

**DOI:** 10.3390/ijerph192416633

**Published:** 2022-12-11

**Authors:** Faris Yahya I. Asiri, Marc Tennant, Estie Kruger

**Affiliations:** 1 Department of Preventive Dental Sciences, College of Dentistry, King Faisal University, Al-Ahsa 31982, Saudi Arabia; 2 International Research Collaboration—Oral Health and Equity, School of Human Sciences, The University of Western Australia, Perth, WA 6009, Australia

**Keywords:** oral health, barriers to oral health, accessibility to oral health, individuals with disabilities, special care

## Abstract

Various studies have indicated that persons with special needs may face several barriers to dental treatment, which increases the prevalence of oral diseases in this population. Moreover, these studies suggested that Saudis with special needs have a higher prevalence of oral diseases, such as dental caries and periodontal disease. The aim of this review is to synthesize evidence regarding the current status, trends in oral health behaviors, and oral health care utilization among these individuals, as well as to assess the quality of the literature. Furthermore, this review seeks to recommend directions for future research and oral health care policymaking. An electronic search was conducted using the following databases and registers: PubMed/Medline, Embase, ISI Web of Science, Scopus, ClinicalTrials.gov, and CENTRAL. Gray literature, which included conference proceedings and unpublished literature, was searched via the library services and Google/Google Scholar, and the quality of studies was assessed using the AXIS scale for cross-sectional studies. A total of 38 studies were included in this review, with the majority of the studies graded as ‘low’. Within the limitations of this study, it can be concluded that individuals with special needs have limited access to oral health care, poor oral health status, and a general lack of awareness in regard to oral health. Therefore, nationwide surveys should be carried out to ascertain the actual extent of the oral inequities among individuals with special needs.

## 1. Introduction

The contemporary concept of disability is that individuals with disabilities are differently abled persons who have a different capacity to perform functions [1]. Disability can be apparent, such as physical and sensory, or intellectual and cognitive. It usually affects a person’s movement, vision, thinking, hearing, learning, speech, communication, memorizing, and social interactions [2,3]. The World Health Organization (WHO) and World Bank 2011 reports have stated that 15% of the global population is living with some form of disability, of which a majority (80%) belongs to the developing world [4]. It has been indicated that persons with special needs may face several barriers to dental treatment, which increases the prevalence of oral diseases in this population [5]. Individuals with special needs have poor oral health and face oral inequity more frequently than those without disabilities [6]. Additionally, developmental disorders, such as Down syndrome, may alter immune responses, making the individual more susceptible to periodontal disease and peri-implantitis [7]. Efforts have been made internationally to educate and train dentists to treat individuals with special needs [8]. In Saudi Arabia, it has been estimated that 2.9% of the total population have an extreme form of disability [9]. Moreover, studies suggest that Saudis with special needs have a higher prevalence of oral disease, such as dental caries and periodontal disease [10].

Several barriers to the oral health care of individuals with special needs have been identified, including distance, lack of trained specialists, lack of awareness or education of the caregivers, and costs of the treatment [11,12,13,14,15]. Patients with intellectual disabilities may underestimate the importance of oral health care and may not know how to access oral health care opportunities. Moreover, the dental treatment of individuals with special needs may take longer and may require additional personnel and sedation or anesthesia modalities. Over the last few years, several studies and reviews have been published on the oral health of Saudis without disabilities [16,17]. However, to date, no systematic review has been published that summarizes the status of oral health, oral health behaviors, and oral health care utilization among persons with disabilities in the Saudi population. Therefore, this review aims to synthesize evidence regarding the current status, trends in oral health behaviors, and oral health care utilization among these individuals, as well as to assess the quality of the literature. Furthermore, this review aims to recommend directions for future research and oral health care policymaking.

## 2. Materials and Methods

### 2.1. Focused Question

Utilizing the Participants, Intervention, Control, and Outcomes (PICO) principal as defined in the Preferred Reporting Items in Systematic Reviews and Meta-analysis (PRISMA), the following focused question was constructed: ‘What is the oral health status, oral health behaviors, and oral health care utilization among persons with disabilities, and is it similar to those without disabilities in Saudi Arabia? Is the status of oral health, oral health behaviors, and oral health care utilization among persons with disabilities similar to those without disabilities in Saudi Arabia?’

### 2.2. Eligibility Criteria and Literature Search

An electronic search was conducted on the following databases and registers: PubMed/Medline, Embase, ISI Web of Science, Scopus, ClinicalTrials.gov, and CENTRAL. Gray literature, which included conference proceedings and unpublished literature, was searched via the library services and Google/Google Scholar. All clinical studies focused on the oral health status and/or self-reported oral hygiene practices (tooth brushing, flossing, fluoride use, smoking, and sugar consumption) and/or studies that assessed oral health care utilization by evaluating the dental attendance status and barriers encountered while accessing dental care services. Exclusion criteria for the study were non-English letters to the editors, commentaries, and studies (MeSH keywords are provided in Supplementary File S1). Moreover, the reference lists of the included articles were searched for additional studies. Two investigators independently conducted the literature search and any disagreements were resolved through discussion. Furthermore, we calculated the inter-examiner reliability (Kappa) score.

### 2.3. Data Extraction

The data from each included study were extracted using pre-calibrated data forms by two investigators independently using the Covidence platform. The data were validated by a subject matter expert. Briefly, in addition to the PICO information, the following data were extracted: study author and year, gender, intervention/observed groups, target population and the actual included population, the type and number of centers in which the samples were studied, the assessed variables, and the overall outcomes (qualitative and/or quantitative). The data from the forms were extracted into Microsoft Excel prior to validation. A qualitative meta-analysis was not feasible due to the heterogeneity of the studies.

### 2.4. Quality Assessment

The quality of the studies was determined by using the critical appraisal tool to assess the quality of cross-sectional studies (AXIS) [18]. Briefly, various aspects of introduction, methodology, results, discussion and other sections of studies were assessed. Any disagreements were solved by discussion.

## 3. Results of the Literature Search

**Figure 1:**
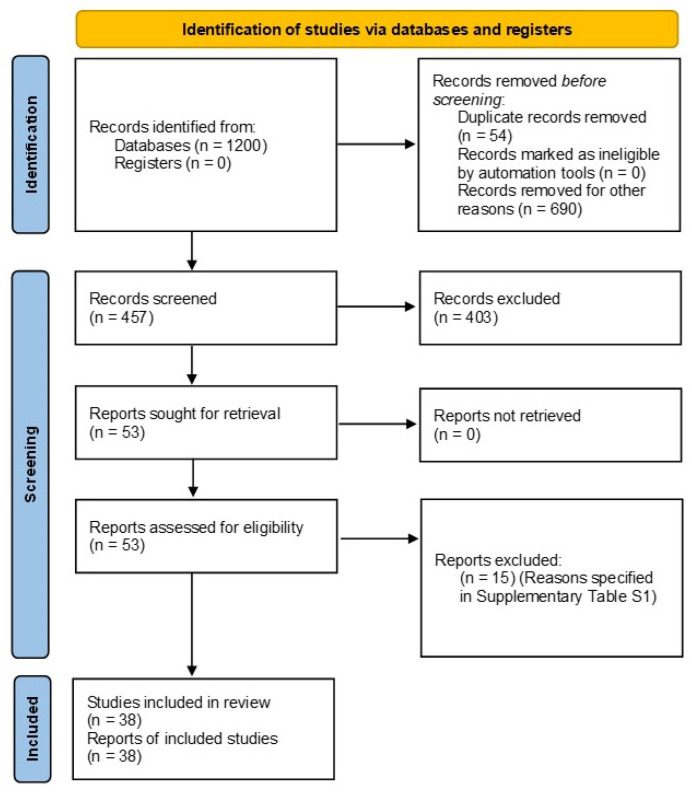
Prisma flow diagram of the search methodology employed for this review.

The initial literature search resulted in 1657 items. After the removal of 1200 items, the titles and abstracts of 457 articles were read to determine eligibility. Ultimately, the full texts of 53 research articles were downloaded for potential inclusion. Of these, 15 articles were excluded and are listed in Supplementary Table S1, along with the reasons for their exclusion (Supplementary Table S1). Therefore, 38 studies were included in this review [10,11,12,13,14,19,20,21,22,23,24,25,26,27,28,29,30,31,32,33,34,35,36,37,38,39,40,41,42,43,44,45,46,47,48,49,50,51]. No additional studies were found upon hand searching or searching the gray literature. The inter-examiner reliability (Kappa) score was calculated as 0.89. The literature search process is presented in Figure 1.

## 4. General Characteristics of Studies

Thirty-five studies were cross-sectional [10,11,12,13,14,19,20,21,22,23,24,25,26,27,29,30,31,32,33,34,35,36,37,38,39,40,41,42,43,44,46,47,48,50,51], one was a longitudinal study [45], and two were retrospective studies [28,49]. Twenty-two studies were conducted in or near Riyadh [10,11,13,14,19,20,21,22,23,24,26,27,28,29,32,33,34,35,38,46,49,51]. Populations with special needs in the following cities were also studied: Al-Hofuf [11], Makkah [25], Khobar [30,50], Dammam [12,30,34], Qatif [30,39,44], AlMadinah Munawwarah [31], the Abha/Albaha province [34,36], Jeddah [37], Taif [40,42], Al-Kharj [41,45], the Aseer region [43], Makkah [47], Al-Qassim [48], Dhahran [50], Anak [50], Dareen [50], UmulSahik [50], and Al-Nabia [50]. In 13 studies, the target population was selected from multiple institutions or schools [10,12,13,20,21,26,27,30,33,39,43,44,46], while in the remaining studies the sample population was either from a single institution or the exact number was not stated. The sample sizes of included studies ranged between 23 and 819 participants [10,11,12,13,14,19,20,21,22,23,24,25,26,27,28,29,30,31,32,33,34,35,36,37,38,39,40,41,42,43,44,45,46,47,48,49,50]. Overall, 942 participants with Down syndrome, 1070 with autism spectrum disorder, 564 with cerebral palsy, 466 with a visual impairment, 785 with a hearing and/or speech impairment, 656 with intellectual disabilities, 111 with learning disabilities, 30 with epilepsy, 221 that were medically compromised, and 128 with unclassified disabilities were included. Therefore, in this systematic review, data from 4441 individuals with special needs were included [10,11,12,13,14,15,19,20,21,22,23,24,25,26,27,28,29,30,31,32,33,34,35,36,37,38,39,40,41,42,43,44,45,46,47,48,49,50]. The ages of the included participants ranged between 3 and 60 years, with the majority of the studies focusing on children with special needs. Four studies did not report the gender of the included participants [12,34,43,46], and in four other studies, no female participants were included [30,36,38,45]. The complete record of the general characteristics is provided in Table 1.

## 5. Overall Outcomes of Studies

The most frequently measured oral health outcome was dental caries [12,14,24,25,29,30,31,32,35,36,37,38,41,42,43,45,46,47,48,49]. The included studies showed a high prevalence of dental caries in the majority of the population with special needs or disabilities. It was also observed that in the majority of individuals with special needs, there was a high prevalence of gingival and periodontal disease [28,29,35,39,41,43,50].

Three studies showed a significantly higher prevalence of gingival inflammation and dental caries in children with special needs (ASD, hearing impairment, and visual impairment) [28,29,30] and that children with visual impairment, hearing impairment, and cerebral palsy are more likely to experience dental trauma [20,33,40]. Moreover, there was a higher need for orthodontic treatment in patients with Down syndrome (66–81.9%) due to Class III malocclusions [19,22,23,43]. Children with intellectual disabilities had a higher prevalence of dental caries than children with blindness or deafness [24], and people with intellectual disabilities and cerebral palsy had a higher risk and prevalence of dental caries, compared to people with other physical or mental disabilities [25].

- Oral health behavior:

Overall, there was a low frequency of tooth brushing (less than twice a day) and flossing among the participants [11,25,26,27,30,31,34,35,50]. When dietary habits were studied, sugar consumption was high and was associated with a high caries rate [12,25,27,42]. In one study, sugar consumption was low and this was associated with the overall positive parental attitude towards oral health [39]. One study showed that obesity may act as a co-factor in aggravating the dental caries prevalence in children with special needs [42]. In the studies included, there was variability in the brushing habits of persons with disabilities. Murshid et al. observed that up to 60% of participants with ASD could not brush their teeth by themselves [27] and 85.2% needed help during brushing [12]. Moreover, there were statistically significant differences between those with and without visual impairment in terms of tooth brushing (*p* = 0.043) [29], whilst two studies did not find significant differences in brushing habits among persons with and without disabilities [30,50]. In another study, nearly 50% of caregivers or parents helped their children with disabilities with brushing, but this did not have a statistically significant effect on periodontal disease or plaque accumulation. Plaque accumulation and gingival inflammation were significantly associated with reduced brushing practices (=0.004 and <0.0001, respectively) [39]. Ashour et al. observed that dental caries prevalence decreased with the use of fluoridated dentifrice [25].

### Smoking and Substance Abuse

Only two studies explored the effect of smoking on the oral health of persons with disabilities [30,50], and one study found a significantly higher prevalence of periodontal disease in cigarette smokers [50]. No other type of substance abuse was studied.

- Oral health care attendance and barriers:

A study showed that 26.3% of children with special needs have never visited a dentist [13], and the most prevalent reasons for this included: the inability or difficulty of the children to adjust to the environment of the dental clinic (43.4%), parents being too busy with providing medical care to their children (30.1%), and inaccessibility of dental clinics (26.5%). In another study, it was observed that 51.3% of individuals with disabilities had not seen a dentist for more than a year and 84.7% visited a dentist only for emergency treatment. Fear of the dentist was found to be the most common factor impeding dental care to persons with disabilities (52.1%), followed by cost (48.7%), inability to sit in the dental chair (28.2%), transportation issues (26.9%), distance (18.5%), and the lack of skill of the dentist (16.5%) [11]. In other studies, only 28.2% of children with ASD had visited the dentist and this was only in cases of an emergency [26,27]. Al-Qahtani et al. found that 40% of the included sample size of children with disabilities had never visited the dentist before [30]. Two studies also showed that almost half (46.6% to 49.2%) of the participants had never visited the dentist [12,35], and Alsheri et al. found that dental pain was the main reason for visiting a dental clinic [10]. Common barriers to dental care stated by caregivers included distance, transportation difficulties, unsuitable clinic environment care, medical issues, lack of medical insurance or coverage, previous bad experience at the dental clinic, and lack of time [44,51].

## 6. Results of the Quality Assessment

All studies included an adequate objective, justification of the study design, and justification of the results [10,11,12,13,14,19,20,21,22,23,24,25,26,27,28,29,30,31,32,33,34,35,36,37,38,39,40,41,42,43,44,45,46,47,48,49,50,51]. Seven studies included a statistically calculated or a pre-determined sample size [19,20,21,24,29,35,40], nineteen studies adequately described the included target population [12,19,20,21,22,26,27,29,30,34,35,36,37,38,40,42,44,48,50], and thirty studies included an appropriate population base [10,11,13,14,19,20,21,22,23,24,26,27,29,30,31,33,34,35,36,37,38,39,40,41,42,44,45,46,47,48,49,50]. Non-responders were addressed in only two studies [29,37]. Outcomes were adequately measured in 35 studies [10,11,12,13,14,19,20,21,22,23,24,25,27,28,29,30,31,32,33,34,35,36,37,38,39,40,41,42,43,44,45,46,47,48,49,50]. In 17 studies, the piloting or validation of the measurement instrument was carried out [10,11,13,14,21,22,23,24,27,28,30,32,34,35,37,39,40,42,44,45,46,47,48,49,50]. Statistical results were described adequately in 14 studies [12,25,26,27,29,30,34,35,36,37,39,40,42,44]. The basic data were described adequately in 32 studies [10,11,12,13,14,19,20,21,22,23,24,25,26,27,28,29,30,32,34,35,36,38,39,40,41,42,43,44,45,46,47,48,49,50]. The methodology to address the response rate was described in only four studies [15,26,29,41], and non-responder information was provided in only one study [15]. Outcomes were adequately reported in 33 studies [10,11,13,14,20,21,22,23,24,25,26,27,28,29,30,32,33,34,35,36,37,38,39,40,43,44,45,46,47,48,49,50]; in the discussion section, 37 studies justified the results adequately [10,11,12,13,14,19,20,21,22,23,24,25,26,27,28,29,30,31,32,33,34,35,36,37,38,39,40,41,42,43,44,45,46,47,48,49,50] and limitations where identified in 16 studies [12,22,25,26,27,28,29,30,31,33,34,36,39,40,51]. Funding information was provided in 11 studies [12,22,29,30,33,36,37,39,40,41,48,51]. Funding or ethical information was provided in 23 studies [11,13,14,21,22,23,24,25,26,27,28,29,30,31,32,33,34,35,36,37,39,40,42,44]. Eighteen studies received a grading of ‘low’ [10,11,13,14,23,24,28,31,33,38,41,43,45,46,47,48,49,50], the same number of studies received an overall grade of ‘moderate’ [12,15,19,20,21,22,25,26,27,30,34,35,36,37,51], and three studies received a grade of ‘high’ [29,39,40]. The detailed results of the quality assessment are presented in Table 2.

## 7. Discussion

Children and young adults with disability and special education needs are known to have poorer oral health and greater unmet dental needs, compared to that of the general child population [52]. In addition to receiving high-quality clinical care, improving the oral health of people with disabilities necessitates that they have adequate access to dental care. Before recommending solutions, an awareness of the obstacles that people with disabilities face when trying to access dental treatment is required.

The government of Saudi Arabia has planned to modernize health care by 2030. The main aim of this agenda is to promote healthy lives. An action plan specifically for the aforementioned program is the “Health Sector Transformation Program” [53]. Its goal is to improve the accessibility of health care services to the entire population and deliver people-centered services by providing quality services along with disease prevention and promotion [54]. Therefore, the goal of this systematic review is to summarize and analyze the literature that has been published, focusing on oral health care (including the barriers, access, and utilization of oral health services) among people with special needs in Saudi Arabia. Additionally, this study also aims to describe the current oral health status of people with disabilities. 

This study observed that the majority of research participants had poor oral hygiene practices and were dependent on caretakers to maintain proper oral hygiene and access oral health care services. As a result, caregiver training and education should be considered to support preventative approaches to oral disease [55]. Previous studies have indicated that training health care workers to treat individuals with special needs improves their ability to provide health care and may improve outcomes, when compared to those providers with no such training [56].

Over the last few decades, there have been significant advances in medical care for persons with special needs, leading to greater life expectancy. Therefore, oral health provisions for people with special needs need to be tailored and made accessible. Dental care is recognized as an integral component of the basic health requirements of children and adults with special needs [57]. Globally, one in every ten individuals live with disability, and the majority lack access to dental care [58]. Although disability in Saudi Arabia is a major concern, both socially and economically, there is very limited literature available to analyze the situation in detail [59].

Over the years, approximately 4–8% of Saudi Arabia’s population has become disabled, but only a small percentage receives proper health care services [44]. In Saudi Arabia, according to the General Authority for Statistics, 2.9% of the population is living with an extreme form of disability [9]. Therefore, there is a need a for comprehensive guidelines and policy to make dental care accessible for Saudis with special needs. Results from this review suggest that there are several gaps in the research that need to be overcome to achieve this goal. Arguably the biggest deficiency in the literature concerning dental care of the Saudi population with special needs, is the lack of national-level surveys assessing access to dental care, oral health status, and knowledge of caregivers [60,61]. This is indicated by more than two-thirds of studies being conducted in one city, Riyadh [10,11,13,14,15,19,20,21,22,23,24,25,26,27,28,32,33,35,38,46,49]. Participants from smaller cities and towns need to be studied to gauge a more representative view of the national situation. The lack of representation is shown by the majority of studies conducted in single institutions. Furthermore, the majority of the studies that did carry out multi-center surveys, did not include individuals with special needs from mainstream schools. Since special needs schools are more likely to provide an oral and systemic health care regimen personalized to the disabilities of the patient, the outcomes of the studies could have been influenced. Future studies should include both individuals with special needs living or studying in specialized institutes and those studying in mainstream schools.

An important observation made in this review are the barriers and difficulties faced by individuals with special needs in receiving dental care. Surveys in rural areas would reveal additional data regarding issues, such as distance and lack of dental clinics. Furthermore, more detailed studies should be conducted regarding the skills of general dental practitioners in treating individuals with special needs. We also recommend establishing continuing education or specialist programs that train dentists in special care dentistry. A glaring omission in the literature is a lack of studies focusing on adults with special needs, with only three studies [34,38,50] focusing on adults. With increasing age, psychomotor issues, lack of parental assistance, and poor oral hygiene, adults with special needs are expected to have a higher prevalence of periodontal disease and dental caries, which can add to the difficulties encountered by these individuals. Therefore, specialized dental programs aimed at adults with special needs should be initiated.

There are several recommendations that could be made to attempt to improve the oral health care of individuals with special needs in Saudi Arabia. Firstly, there needs to be specialized training programs in special care dentistry. Similar training for dental therapists, hygienists and assistants should be conducted. The oral health care for children with special needs should be accessible. One way to do this would be to create mobile dental hygiene services that can visit special needs centers. Oral health education programs need to be implemented in special needs centers and among children with special needs in mainstream schools. Dental outreach programs focusing on the population with special needs should be mandated among dental students. Doing so may not only make dental care accessible for patients but also familiarize future clinicians with providing dental care to these patients. Special care dentistry should be emphasized in the dental curriculum, dental offices and clinics should be designed appropriately to cater for people with special needs (focus on ramps and elevators for ease of access), and conscious sedation should be available and used where appropriate. To date, there has not been a guideline published in regard to the provision of general anesthesia or conscious sedation to patients with special needs and disabilities.

The included studies had several limitations. As shown in the quality assessment results, the majority of the studies were graded as ‘low’. The majority of the studies did not have a control group comparing the oral health issues of individuals with special needs to those without them. Furthermore, the majority of the studies failed to carry out randomization of the included sample size. Additionally, due to ethical reasons, it is difficult to blind patients, participants, or caregivers. Therefore, performing randomized controlled trials would be considered unethical, which adds a further limitation to future studies. The authors plan to conduct systematic reviews, focusing on oral health status in Saudi individuals with specific disabilities and special needs.

## 8. Conclusions

Within the limitations of this study, it can be concluded that individuals with special needs have limited access to oral health care, poor oral health status, and a general lack of awareness about oral health. Furthermore, the differences in the age and overall health of individuals with special needs make it difficult to present an overall assessment of oral health behaviors and oral health care utilization among persons with disabilities in Saudi Arabia. Standardized nationwide surveys should be carried out to ascertain the actual extent of the oral inequities among individuals with special needs.

## Figures and Tables

**Table 1:** List of studies about oral health status, oral health behavior, and oral health care utilization.

Study (Author, Year)	Study Design and Aims	City and/or Province	Study Setting (*n*, Number of Centres if Provided)	Target Population (Sample Size, *n*)	Special Needs Included (*n*, if Applicable)	Ages (Range, Mean)	Gender, Female (n, %)	Responders/Subjects Included/Groups (n)	Variables Measured/Dental Indices	Main Oral Health Outcomes and/or Observations
AlSarheed et al., 2003	Cross-sectional	Riyadh	schools (n = 2)	781	VI, HI	11–16 years	n = 423, 54.1%	Group 1: VI (n = 77) Group 2: HI: (n = 210) Group 3: Healthy controls (n = 494)	OH status (IOTN) (DHC) index aesthetic component (AC).	A total of 21.8% of HI, 65% of VI, and 18.7% of controls needed orthodontic treatment. Patients with special needs had a higher need of orthodontic treatment (with males with VI impairment requiring the highest).
AlSarheed et al., 2003	Cross-sectional	Riyadh	schools (n = 2), (1 = VI) and (1 = HI)	781	VI, HI	11–16 years	n = 423, 54.1%	Group 1: VI (n = 77) Group 2: HI: (n = 210) Group 3: Healthy controls (n = 494)	OH status (TDI index).	Both HI (11.4%) and VI (9%) had higher incisor trauma rate than controls (6.7%). HI statistically higher than controls (*p* < 0.05).
AlSarheed, 2004	Cross-sectional	Riyadh	Special needs schools vs. mainstream schools (n = not stated)	781 parents	VI, HI	11–16 years	n = 423 (54.1%)	Parents of 3 groups: Group 1: VI (n = 77) Group 2: HI: (n = 210) Group 3: Healthy controls (n = 494)	OH status (IOTN).	Parent attitude to children’s teeth; 77% of VI parents, 47% of HI parents, and 62.5% of control parents believed their children’s teeth were maligned. Parents attitude towards OTN of their children: 31.1% of VI parents, 23.6% of control parents and 17.9% of HI parents believed their children were concerned with their dental appearance (VI vs. HI (*p* < 0.05)). Parents attitude towards OH and OT: Approximately 25% of parents believed that oral hygiene would be difficult during OT (no difference between groups). Approximately 50% parents believed that OT would be difficult to commence.
AlKawari, 2021	Cross-sectional	Riyadh	Special needs institutes (n not stated)	n = 23 children with DS	DS	10–14 years	74%	N/A	OH status IOTN-DC. Angle’s classification.	A total of 81.9% of children with DS needed OT, with the majority having severe malocclusion. A total of 59.1% had Angle Class III malocclusion, and 36.4% had Angle Class I.
Alkhadra et al., 2017		Riyadh city	Rehabilitation centers (*n* = 5)	200 children (100 DS and 100 AD)	(DS) and AD	6 to 14 years	n = 69 DS 34 AD 35	N/A	OH status Angle’s classification.	Malocclusion: DS: 66% (mostly Class III); cross bites, 48% AD: 3–4% (mostly class I).
Qahtani and Wyne, 2004	Cross-sectional	Riyadh	Special needs school	219 children	VI, HI, ID	6–7 years and 11–12 years	n = 219 (100%)	Group 1: VI (n = 12), 6–7-year-olds Group 2: HI (n = 23), 6–7-year-olds Group 3: ID (n = 32), 6–7-year-olds Group 4: VI (n = 17), 11–12-year-olds Group 5: HI, (n = 57), 11–12-year-olds Group 6: ID (n = 109)	OH status (DMT/dmft).	Aged: 6–7-year-olds VI dmft = 7.58 ± 2.02 (decay component: 6.33 ± 2.74); DMFT: 1.67 ± 1.67 (only decay component) Caries prevalence: NR HI dmft = 7.35 ± 3.82 (decay component: 7.09 ± 3.55); DMFT: 0.87 ± 1.25 (only decay component) Caries prevalence: 95.7% DI dmft = 8.00 ± 4.1 (decay component: 2.39 ± 1.64); DMFT: 3.00 ± 2.11 (2.39 ± 1.64) Caries prevalence: 93.9% 11–12-year-olds VI dmft = 1.00 ± 1.9 (only decay component); DMFT = 3.80 ± 2.67 (decay component: 3.76 ± 2.66) Caries prevalence: 88.2% HI dmft = 2.11 ± 2.53 (decay component: 1.9 ± 2.37); DMFT: 5.12 ± 3.45 (decay component: 4.79 ± 3.14) Caries prevalence: 93% DI dmft: 3.2 ± 3.18 (decay component: 3.16 ± 3.05); DMFT: 5.81 ± 2.95 (decay component: 5.16 ± 2.62) Caries prevalence: NR Children with DI had the worst oral hygiene.
Wyne, 2007	Survey	Riyadh	centre (n = 7)	315 parents	DS (n = 117), CP (n = 106), ID (n = 54), Others (n = 38)	36.3 years (parents); 7.7 years (children)	n = 245 (88.2%) mothers; 39% female children	<5 years 6–10 years >11 years	OH care utilization. Dental visits and barriers.	Only 17.1% of children had visited the dentists by age 7. A total of 26.3% of children had never visited a dentist before. The reasons for not visiting a dentist: A total of 43.4% was due to child’s behavior difficulties. A total of 30.1% was because parents are too busy in the medical care of their child. A total of 26.5% was due to the inaccessibility of dental services for children with disabilities. A total of 73.7% had already visited a dentist for 22.0%, their last visit was due to pain in teeth, for 32.7% it was for a follow-up appointment, and for 45.3% their last dental visit was their first ever dental check-up. Parents with a higher education level had a more positive attitude towards dental visits than those with a lower education level (*p* < 0.05).
Sharifa and Al-Shehri, 2012	Survey	Riyadh and Al-Hfouf	Centres	119 caregivers	Autism (n = 2), DS (n = 22), ID (n = 45), LD (n = 41), Others (n = 9)	Between 16 and 60 years	Caregiver 63% disabled 75.2%	1–10 years 11–15 years 16–20 years >21	OH behavior (tooth brushing). OH care utilization (dental visits/barriers).	A total of 41.2% could not brush independently. A total of 32.8% could not brush their teeth at all. A total of 51.3% had not visited the dentist in the last year. A strong association was found between caregivers’ level of education and tooth brushing (*p* = 0.046). Barriers: Fear of the dentist (52.1%). Cost (48.7%). Unable to sit in dental chair (28.2%). Transportation (26.9%). Distance (18.5%). Skills of dentists (16.8%). A total of 54.6% required dental treatment, while 30% did not need treatment; 46.2% of individuals with disabilities had difficulty in getting dental care in their community.
Ashour et al., 2018	Analytical cross-sectional study	Makkah	Schools	272 Females	ID (n = 79) AD (n = 41) CP (n = 17) DS (n = 52) DB (n = 61) others (n = 25)	Age group: 6–11 years, 12–17 years	272 Females	Age group: 6–11 years, 12–17 years	OH status (dmft/DMFT). OH behavior (sugar consumption, toothbrushing, and fluoridated toothpaste).	The overall prevalence of caries was 56.7% and the mean caries score (dmft = 3.9, DMFT = 3.2) for the entire study population was high. The caries prevalence was high among intellectually disabled children (77.2%), autistic children (65.8%), and Down syndrome children (61.5%). Regression analysis showed a strong association between intellectually disabled children (adjusted OR = 2.2), autistic children (adjusted OR = 1.2), Down syndrome children (adjusted OR = 1.2), and caries prevalence. A total of 21% of the children were overweight and 21.8% were obese. Mean BMI was 20.2 (2.8). When adjusted for covariates, the logistic regression model showed strong association between caries and obesity (adjusted odds ratio = 2.9; 95% CI = 1.2–4.9). Sugar consumption: 203 answered YES. Tooth brushing frequency (64) ≥ 2 times/day. Fluoridated toothpaste: n = 148 answered YES. Children who consume sugar have a 1.9 times greater risk of developing caries. Children brushing their teeth ≥1 per day had a 2.7 times greater risk of dental caries.
Murshid 2014	Cross-sectional	Riyadh	Centers (n = 3)	450 parents of children with ASD	ASD	3–14 years	24.1%	n = 344 parents of children with ASD	OH behavior (tooth brushing). OH care Utilization (dental visits).	Majority (61.3%) of children are not able to brush teeth themselves. Only 29.1% of children brushed twice a day. Time of first dental visit only in an emergency was 28.2%, necessary only at signs of pain or dental problems. Only 2% of parents thought it should be during the 1st year after the child’s birth.
Murshid 2014	Cross-sectional study	Riyadh	Special needs centers (n = 3)	450 parents of children with ASD	ASD	3–14 years	24.1%	n = 344 parents of children with ASD	OH behavior (sugary food consumption, soft drink consumption, and tooth brushing).OH care utilization(dental visits and type of dental treatment that had been utilized).	A total of 70.9% preferred food that is high in sugar. A total of 96.7% consumed soft drinks regularly. A total of 34.0% brushed their teeth once a day, while 29.0% brushed twice a day, and 28.8% brushed on an irregular basis. Dental visit: A total of 51.5% had no previous dental visits or dental treatment. A total of 10.1% were using nitrous oxide. About 25% received treatment under general anesthesia. A total of 48.5% used different behavioral management techniques for dental treatment. A total of 48.5% of the children had dental problems treated.
Diab et al., 2016	Retrospective	Riyadh	Special needs school	50 children	ASD	8.5 years (4–15 years)	n = 26	n = 50 children with ASD n = 50 children without ASD	OH status (GI, PI, salivary pH, and salivary buffering capacity).	Children with ASD have higher gingival inflammation (*p* < 0.005), poor oral hygiene (*p* < 0.005), and lower salivary pH (*p* < 0.05), when compared to children without ASD.
AlSadhan et al., 2017	Cross-sectional	Riyadh	VI school vs. Mainstream school	n = 162 children	VI	9.81 years (6–12 years)	n = 162 (100%)	n = 79 children with VI n = 83 children without VI	OH status (DMFT/DMFS)/(dmft/dmfs) (OHI) (PI) (GI). OH behavior (tooth brushing). OH care Utilization (dental visits).	Children with VI had poorer DMFS (*p* < 0.05), lower OHI (*p* < 0.001), and poorer systemic health (*p* < 0.005). Tooth brushing: Only 78.5% of the VI children and 90.4% of children without VI; the difference was statistically significant (*p* = 0.043). A total of 71% of the children without VI had been to the dentist, compared with 54.5% of the VI children (*p* = 0.028).
Al-Qahtani et al., 2017	Cross-sectional	Eastern Province, cities of: Khobar, Dammam, and Qatif.	Schools (n = 7).	n = 327	Deaf, HI	NR	0%	n = 109 children with HI n = 218 children without HI	Oral H status (Dental caries) (DMFT/DMFS). Oral H behavior (brushing, flossing). Oral H care utilization (dental visits).	More than 97% of the deaf and 81.8% of the HI in the 12–14 age group had decay, compared to 64.9% in the controls (*p* = 0.009). The differences between the children with HI and children without HI were statistically significant (Tukey’s test, *p* = 0.005). More severe forms of caries were common in the deaf children (34.9%) than in the children with HI (30.4) and children without HI (16.8%). The overall mean DMF/S for all children was 10.03, greater than the finding in Indian and Kuwaiti adults with special needs. The 12–14-year-old group was statistically significant for the “D” component and the “DMF/S” (*p* = 0.005) and (*p* = 0.003), respectively. The difference was also significant for the “F” component for the same disability and age groups (*p*-value of 0.003). The DMF/S score (prevalence of dental caries) increased with age in all the groups. A total of 10% do not brush, and 88% do not floss. Around 40% of the deaf students reported never visiting a dentist before.
Alhazmi, et al., 2014		Al Madinah		n = 80 children	VI	7–24 years	(29 female)	64 children	OH status (dmft or DMFT). ➢ Plaque and calculus index.OH behavior (brushing, flossing).	Caries prevalence of the VI children is 95.16%, which is very high. Low DMFT/dmft 0.24/0.59 and more than 2/3 have equal or greater than 1–2 soft debris accumulation. There is no significant difference between the mean of DMFT/dmft for both male and female genders and the mean of plaque index. A total of 85.9% brush their teeth (43.1% brush their teeth twice per day). Independent in brushing: A total of 62% brush teeth without any help. Dental floss: 10.9% used it, and 89% never used it before.
Wyne et al., 2017	Cross-sectional	Riyadh	Special needs school (n = 2)	n = 52 children	CP	6.3 years (3–10 years)	38.5%	n = 52 children with CP	OH status (DMFT + dmft).	A total of 98.1% of children with CP had dental caries (DMFT + dmft: 9.98 ± 3.99).
Al-Sehaibany 2018	Cross-sectional	Riyadh	Special needs schools (n = 3) vs. mainstream schools (n = 3)	n = 514 children	AD	4.15 years	F to M ratio: 1:2.3	n = 257 children with ASD n = 257 children without ASD	OH status clinical examination TDIs.	Prevalence of TDIs in children with ASD (25.7%) is significantly higher than without ASD (16.3%) (*p* < 0.05).
Kotha et al., 2018	Cross-sectional/survey	Dammam	Special needs schools (n = 3)	NR	AD	5.8 years	NR	Children with ASD (n not stated)	OH status (dmfts). OH behavior (tooth brushing, sugar, and soft drinks consumption). OH care utilization (dental visits).	Frequency of sugar intake between meals increased dental caries occurrence. - Mean (dmfs) for males was 3.42 and 4.55 for female. - A total of 85.2% needed help during brushing; 73.8% were helped by their mothers. - Dental visits: 49.2% had never seen a dentist before and many were only taken to the dentist if they had a problem—36.1% attended the dentists. No significant effect of OH and level of parents’ education on dental caries.
Mustafa et al., 2018	Cross-sectional/analytical survey	AlKharj, Riyadh, Dammam, Abha	Special needs schools and institutes (*n* not stated)	n = 240 children and adults	HI, SI	15–30 years	NR	N/A	OH behavior (brushing, flossing). OH care utilization (dental visits).	A total of 69% were not aware of the right way of brushing. Majority did not use dental floss. Lack of awareness of OH and dental treatment among individuals with HI and SI. −A total of 72% of the participants had never visited a dentist before.
AlKahtani et al., 2019	Cross-sectional	Riyadh	Teaching dental institute (n = 1)	n = 146	HI	18–21 years	105 (71.9%)	N/A	OH status (DMFT) (GI) (PI) simplified (OHI-S). OH behavior (tooth brushing). OH, care utilization (dental visits).	High dental caries experience and need for dental treatment in the majority of adults with HI. Oral hygiene was fair. n = 55 (37.7%) brushed their teeth twice daily. n = 68 (46.6%) visited dentist in the last 6 months. A total of 40 (55.6%) of 18–21 years, 19 (26.4%) 22–25 years, and 13 (18.1%) in >25 years were in need of preventive caries with statistically significant differences (*p* = 0.036).
AlZahrani et al., 2019	Cross-sectional (mixed methods)	Albaha province	Special needs school (n = 1)	n = 92 children (only male); oral control (n = 46); ILD (n = 46)	ID	12–16 years	0	ID = 92 oral control (n = 46) ILD (n = 46)	OH status DMFT.	High prevalence of dental caries, dental pain, and poor oral health in majority of children with ID.
Alaki and Bakry 2012	Cross-sectional (mixed methods)	(Jeddah)	Children visiting the hospital dental clinics at King Abdelaziz University (KAU),	86 children	ID	Age: 12–16 years		ID = 33 Without ID = 53	OH status (DFT/dft).	DFT score was significantly higher in participants with ID (*p* = 0.04). Higher ‘D’ component compared to that in children without ID (*p* = 0.03). DFT score was higher in healthy children (*p* = 0.04) with higher ‘d’ component (*p* = 0.05). DFT/dft scores did not include the (M/m) component. ID group had significantly more salivation (*p* = 0.01), and more put their hands inside their mouths (*p* = 0.003).
Gufran et al., 2019	Cross-sectional (analytical)	Riyadh	Special needs school (n = 1)	n = 81 young adults and adults	DS	16–40 years	0	N/A	OH status. (DMFT) (GI) (PI).	Poor periodontal health and high prevalence of dental caries and PI in the majority of males with DS. No association of age with GI. Younger subjects had higher PI (*p* < 0.001).
AlHumaid et al., 2020	Cross-sectional (analytical)	Eastern Province: Dammam, AlKhobar, Dhahra, Al-Qatif	Special needs schools (n = 13)	n = 75 children with ASD	ASD	6–18 years (10.8 years)	F: M ratio = 1:1.27	N/A	OH status (DMF) (GI) (PI). OH behavior (tooth brushing, flossing, sugar consumption).	The prevalence of dental caries in primary dentition was 76% and 68% in the permanent teeth, with a mean of 0.85 ± 1.9 and 1.03 ± 2.9, respectively. A total of 31 had gingival disease, mean gingival index was 1.03 ± 0.88, Mean plaque index was 0.95 ± 0.43 *n* = 17/22.7% did not brush. A total of 61.3% did not floss. A total of 18 (24%) always consumed sugar. Positive parental attitude resulted in lower sugar intake and better oral health.
Basha et al., 2021	Cross sectional	Taif	Special needs schools	n = 350 children with MD	Obesity, ID, ASD, CP, HI, DS, MD ID (n = 121) A (n = 74) CP (n = 40) DS (n = 65) DB (n = 30) MD (n = 20)	6–16 years	n = 219	6–11 years n = 118) 12–16 years n = 232)	OH status (TDI).	A total of 23.1% of children with special needs had TDIs. Children with obesity had a high prevalence of TDIs (30.3%). Children with CP were 3.18 times more likely to experience TDIs than other disabilities.
Shah et al., 2015 ([41])	Cross-sectional	Al-Kharj	Special needs center	*n* = 80	Learning Disability—22 Epilepsy—14 Cerebral Palsy—26 Down syndrome —4 Behavior Disorder—5 Attention Deficit Hyperactivity disorder (ADHD)—2 Multiple Diagnosis-7	16–50 years			OH status (DMFT/ (DMFS) Clinically examined periodontal conditions. Retained teeth cross-tabulation.	Mean DMFT: a mean DMFT of 3.75, slightly higher than 3.34 of the Saudi population. The majority presented with poor oral hygiene and a higher periodontal treatment complexity. Retained deciduous tooth: (25%) 20 patients had at least one retained deciduous tooth. Higher numbers were found in those with a learning disability, multiple diagnoses, and Down syndrome.
Mohamed et al., 2021 ([42])	Cross-sectional	Taif	NR	n = 400 children with MD	ASD (n = 107), CP (n = 43), DS (n = 70), ID (n = 123); HI/VI (n = 33), MD (n = 24)	6–16 years	n = 171 (77.7%)	Underweight/normal weight (n = 214) Obese (n = 186)	OH status (dmft or DMFT) (dmfs or DMFS) OH behavior (sugar consumption and brushing frequency).	Caries prevalence: CP, 76.7%; ASD, 78.5%; DS, 47.1%; ID, 79.7; HI/VI: 66.7%; MD, 79.2%. Obese, 77.9%; Non-obese: 67.3%.
Sandeepa et al., 2021 ([43])	Cross-sectional	Aseer region	Special needs institute (n = 4)	n = 54 children with DS	DS	0–24 years		0–6 years 7–12 years 13–18 years 19–24 years	OH status (DMFT) (OHI) (PI) (PPD) Occlusal abnormalities (visually observed).	The 19–24-year-old individuals with DS had the highest prevalence of PD (71.4%; *p* < 0.05), compared to other age groups. No difference in DMFS among age groups. Females had a higher prevalence of PD and DMFS score, when compared to males. Females had a higher number of cases of poor oral hygiene (66.7%), compared to males (27.3%), which was statistically significant (*p* < 0.05). Malocclusion: All patients had class III skeletal relation. Malocclusion was seen in 42 (75%) and abnormality in the shape, number, or eruption was observed in 30 (53.6%) subjects. Hypoplasia was seen in 19.6% and attrition was seen in 17.9%.
Alfaraj et al., 2021 ([44])	Cross-sectional	Qatif	Special needs schools (n = 8), mainstream schools (n = 3)	n = 700 caregivers	MD	Age of individuals with special needs not reported.		n = 186 caregivers	OH care utilization (barriers).	Difficulties in accessing dental care: Lack of time—54.8%. Unsuitable clinic environment—60.8%. Transportation issues—51.9%. Medical issues—51%. Distance—51%.
Shah et al., 2020 ([45])	A longitudinal study	Al-Kharj:	Special Care School Children	163 children with special needs	VI (n = 8) HI (n = 20) SI (n = 21) DS (n = 33) LD (n = 48) ADHD (n = 8) ASD (n = 20) MD (n = 5)	6–15 years	0	According to their disabilities	OH status (DMFT/dmft) PI.	Total PI of the overall sample = 1.55. The HI and SI group had lower average mean plaque score of 1.02 (SD ± 0.59). This was statistically significant (*p* < 0.05). Plaque scores and mean decayed (D) component were significantly higher in intellectual disabilities, as compared to physical disabilities. There was no significant difference among caries prevalence and decayed components among various groups of disabilities.
Al-Damri et al., 2017 ([46])	Cross-sectional	Riyadh	Special needs centers (n = 3)	NR	DS (n = 100)	8–12 years	NR	DS (n = 100) non-DS (n = 100)	OH status (DMFT) (OHI).	No statistically significant difference was evident between any of the parameters in the control and study group. The results were calculated at 95% confidence level (*p* value = 0.05). After comparison, the values were: D = 0.059, M = 0.090, F = 0.65, and OHI = 0.098.
Alzughaibi et al., 2017 ([47])	Cross-sectional	Makkah	Special needs centers (n unknown)	203 children with DS and non-DS	DS (n = 100)	4–15 years	0	DS (n = 100) Non-DS (n = 103)	OH status DMFT and deft (with salivary amylase, pH, and flow rate).	Deft: DS: 2.72 ± 4.0. Control: 3.88 ± 3.65. *p* = 0.03. DMFT: DS: 2.27 ± 3.9. Control: 1.21 ± 2.08. *p* = 0.02.
AL-Otaibi et al., 2016 ([48])	Cross-sectional	Al-Qassim	NR	206 children with DS and non-DS	DS (n = 121)	6–12 years	Gender of only control mentioned (85 boys)	DS (n = 121) non-DS (n = 85)	OH status DMFT/dmft.	Permanent teeth: DS: 63.9% were caries free. Controls: 80% caries-free. Primary teeth: DS: 80.6. Control: 89.4%. *p* > 0.05.
AlHammad and Wyne 2010 ([14])	Cross-sectional	Riyadh	Special needs center (n = 1)	140 children with CP	CP (n = 140)	3–12 years	41.4%	3–6 years (n = 41) 7–9 years (n = 52) 10–12 years (n = 47)	OH status (DMFT) (OHI).	Caries Prevalence: 98.6%. DMFS: Group 1 A total of 18.8 (±16.3). Group 2 A total of 23.4 (±17.7). Group 3 A total of 20.5 (±14.0). No statistically significant differences (*p* > 0.05) in DMFS scores between the three age groups. No significant difference in DMFS scores between male and female CP children. However, female CP children had significantly higher (*p* < 0.05) filled surfaces than male CP children. Oral hygiene status:The percentage of children with poor OH increased with the age (*p* < 0.05). There was no statistically significant difference between genders in caries and OH. A strong association (*p* < 0.001) was found between OH status and DMFS scores; the children with poor OH had higher DMFS scores
Brown et al., 2009 [49]	Retrospective	Riyadh	Dental clinic/tertiary care center	386 medically compromised and healthy children	Medically compromised (n = 386)	5 years	n = 183	Medically compromised (n = 211) Healthy (n = 175)	OH status (deft index).	Caries prevalence: Medically compromised: 91.9%. Healthy: 84.0%. *p* > 0.05.
Tantawi et al., 2017 [50]	Cross-sectional	Dammam, Qatif, Dhahran, Anak, Dareen, UmulSahik, Al-Nabia, Khobar	Outreach programs	819 adults with and without disabilities	Sensory disabilities (50.9%), motor (33.7%), ID (12.4%), and MD (3%)	32.3 (healthy), 34 (disabled)	n = 401	Special needs (169) Non-special needs (632)	OH status (need for periodontal care). OH behavior (brushing, smoking). OH care utilization (dental visits and treatment utilized).	No significant differences observed between groups in terms of periodontal needs, smoking habits, dental visits, or oral hygiene habits. In need of periodontal care: healthy 66.5% vs. individuals with disabilities 67.3%; overall = 66.8%). Smoking: 27.3% healthy vs. 17.9% in individuals with special needs. Brushing twice or more daily: 54.6% and 55.8%. Dental visits: 46.6% and 46.7%. Professional cleaning: (25.9% and 21.7%.
Al Shehri et al., 2018 ([10])	Cross-sectional	Riyadh	Primary and middle schools (n = 16)	269 children with VI	VI (n = 269)	7–15 years (9.91 ± 2.41)	n = 119 female	None	OH care utilization (dental visits last year and reason).	A total of 28.3% of the females and 36.7% of the males did not receive dental care during the last 12 months. Pain with teeth, gums, or mouth was the main reason for the children’s last visit to the dentist.
Alshihri, Abdulmonem A. et al., 2021 ([51])	Cross-sectional	Riyadh	Societies for special needs (n = 2)	232 mothers	ASD Children (n = 232)	Children between 2.5 and 14 years	29 (20.4%) girls	142 mothers (who are the primary caregivers)	OH care utilization (Previous dental visits and barriers).	A total of 33.8% had not been to a dentist before. A total of 75.4% of children did not have insurance with dental coverage. Barriers reported: Cost (75.4%), finding a dentist (74.6%), uncooperative behavior of child (45.1%). Age did not impact finding a dentist (*p* = 0.429). Having medical insurance and a previous bad experience showed significant effects on the difficulty in finding dental care (*p* < 0.05).

OH, Oral health, VI, visually impaired/impairment; HI, hearing impaired/impairment; DB, deafness or blindness or both; SI, speech impairment; BD, behavior disorder; E, epilepsy; IOTN, index of orthodontic treatment needs; OT, orthodontic treatment; OTN, orthodontic treatment needs; OHI, oral hygiene index; ID, intellectual disability; DMFT, decayed missing filled permanent teeth; dmft, decayed missing filled primary teeth; CP, cerebral palsy; NR, not recorded; N/A, applicable; LD, learning disabilities; AD, autism disorder; ASD, autism spectrum disorder, ADHD, attention-deficit hyperactivity disorder; TDIs, traumatic dental injuries; HCP, health care providers; GI, Modified Gingival Index; PI, plaque index; DS, Down syndrome; CS, cystic fibrosis; MD, multiple disabilities; PD, periodontal disease; PPD, periodontal pocket depth; DVA, dental visual aid. DB-: deafness or blindness or both.

**Table 2:** Quality assessment.

	Introduction		Methodology									Results				Discussion			Others	Overall Quality
Study (Author, Year)	Adequate Objectives of Study	Study Design	Sample Size Justification	Target Population Defined	Appropriate Population Base	Address Non-Responders	Appropriate risk/Outcome Variables Measured	Piloting/Validation of Measurement Instrument(s)	Appropriate Statistics Conducted	Description of Statistics	Basic Data	Response Rate Concerns	Non-Responder Information	Consistency	Adequate Reporting	Justification by Results	Limitations Discussed	Funding	Ethical Approval/Consent	
AlSarheed et al., 2003	Yes	Yes	Yes	Yes	Yes	No	Yes	No	Yes	No	Yes	No	No	Yes	Yes	Yes	No	No	No	Moderate
AlSarheed et al., 2003b	Yes	Yes	Yes	Yes	Yes	No	Yes	No	Yes	No	Yes	No	No	Yes	Yes	Yes	No	No	No	Moderate
AlKawari, 2021	Yes	Yes	No	Yes	Yes	No	Yes	Yes	No	No	Yes	No	No	Yes	Yes	Yes	Yes	Yes	Yes	Moderate
AlKhadra et al., 2017	Yes	Yes	No	No	Yes	No	Yes	No	No	No	Yes	No	No	Yes	No	No	No	No	Yes	Low
AlSarheed, 2014	Yes	Yes	Yes	Yes	Yes	No	Yes	No	Yes	No	Yes	No	No	Yes	Yes	Yes	No	No	No	Moderate
Qahtani and Wyne, 2004	Yes	Yes	Yes	No	No	No	Yes	No	No	No	No	No	No	Yes	No	Yes	No	No	No	Low
Wyne, 2007	Yes	Yes	Yes	No	No	No	No	No	No	No	No	No	No	Yes	Yes	Yes	No	No	Yes	Low
Sharifa and Al-Shehri, 2012	Yes	Yes	No	No	No	No	No	Yes	No	No	No	No	No	Yes	Yes	Yes	No	No	Yes	Low
Ashour et al., 2018	Yes	Yes	No	Yes	No	No	Yes	No	Yes	Yes	Yes	No	No	Yes	Yes	Yes	Yes	No	Yes	Moderate
Murshid, 2014	Yes	Yes	No	Yes	Yes	No	No	No	Yes	Yes	Yes	Yes	No	Yes	Yes	Yes	Yes	No	Yes	Moderate
Murshid, 2014b	Yes	Yes	No	Yes	Yes	No	Yes	Yes	Yes	Yes	Yes	No	No	Yes	Yes	Yes	Yes	No	Yes	Moderate
Diab et al., 2016	Yes	Yes	No	No	No	No	Yes	Yes	Yes	No	Yes	No	No	Yes	Yes	Yes	Yes	No	Yes	Low
AlSadhan et al., 2017	Yes	Yes	Yes	Yes	Yes	Yes	Yes	No	Yes	Yes	Yes	Yes	No	Yes	Yes	Yes	Yes	Yes	Yes	High
Al-Qahtani et al., 2017	Yes	Yes	No	Yes	Yes	No	Yes	Yes	Yes	Yes	Yes	No	No	Yes	Yes	Yes	Yes	Yes	Yes	Moderate
AlHazmi et al., 2014	Yes	Yes	No	No	Yes	No	Yes	No	No	No	No	No	No	Yes	No	Yes	Yes	No	Yes	Low
Wyne et al., 2017	Yes	Yes	No	No	No	Yes	Yes	Yes	Yes	No	Yes	Yes	Yes	No	Yes	Yes	No	No	Yes	Moderate
Al-Sehaibany, 2018	Yes	No	No	No	Yes	No	Yes	No	Yes	No	No	No	No	Yes	Yes	Yes	Yes	Yes	Yes	Low
Kotha et al., 2018	Yes	Yes	No	Yes	No	No	Yes	No	Yes	Yes	Yes	No	No	Yes	No	Yes	Yes	Yes	No	Moderate
Mustafa et al., 2018	Yes	Yes	No	Yes	Yes	No	Yes	Yes	Yes	Yes	Yes	No	No	Yes	Yes	Yes	Yes	No	Yes	Moderate
AlKahtani et al., 2019	Yes	Yes	Yes	Yes	Yes	No	Yes	No	Yes	Yes	Yes	No	No	Yes	Yes	Yes	Yes	No	Yes	Moderate
AlZahrani et al., 2019	Yes	Yes	No	Yes	Yes	No	Yes	No	No	Yes	Yes	No	No	Yes	Yes	Yes	Yes	Yes	Yes	Moderate
Alaki and Bakry, 2012	Yes	Yes	No	Yes	Yes	Yes	Yes	Yes	Yes	Yes	No	No	No	Yes	Yes	Yes	No	Yes	Yes	Moderate
Gufran et al., 2019	Yes	Yes	No	Yes	Yes	No	Yes	No	Yes	No	Yes	No	No	Yes	Yes	Yes	No	No	No	Low
Al Humaid, 2020	Yes	Yes	No	No	Yes	No	Yes	Yes	Yes	Yes	Yes	No	No	Yes	Yes	Yes	Yes	Yes	Yes	High
Basha et al., 2021	Yes	Yes	Yes	Yes	Yes	No	Yes	Yes	Yes	Yes	Yes	No	No	Yes	Yes	Yes	Yes	Yes	Yes	High
Shah et al., 2015	Yes	Yes	No	No	Yes	No	Yes	No	No	No	Yes	Yes	No	Yes	No	Yes	No	Yes	No	Low
Mohamed et al., 2021	Yes	Yes	No	Yes	Yes	No	Yes	Yes	Yes	Yes	Yes	No	No	No	No	Yes	No	No	Yes	Moderate
Sandeepa et al., 2021	Yes	Yes	No	No	No	No	Yes	No	Yes	No	Yes	No	No	Yes	Yes	Yes	No	No	No	Low
Alfaraj et al., 2021	Yes	Yes	No	Yes	Yes	No	Yes	Yes	Yes	Yes	Yes	No	No	Yes	Yes	Yes	No	No	Yes	Moderate
Shah et al., 2020	Yes	Yes	No	No	Yes	No	Yes	Yes	No	No	Yes	No	No	No	Yes	Yes	No	No	No	Low
Al-Damri et al., 2017	Yes	Yes	No	No	Yes	No	Yes	Yes	No	No	Yes	No	No	No	Yes	Yes	No	No	No	Low
Alzughaibi et al., 2017	Yes	Yes	No	No	Yes	No	Yes	Yes	No	No	Yes	No	No	No	Yes	Yes	No	No	No	Low
Al-Otaibi et al., 2016	Yes	Yes	No	Yes	Yes	No	Yes	Yes	No	No	Yes	No	No	No	Yes	Yes	No	Yes	No	Low
AlHammad and Wyne, 2010	Yes	Yes	No	No	Yes	No	Yes	Yes	No	No	Yes	No	No	No	Yes	Yes	No	No	No	Low
Brown et al., 2009	Yes	Yes	No	No	Yes	No	Yes	Yes	No	No	Yes	No	No	No	Yes	Yes	No	No	No	Low
Tantawi et al., 2017	Yes	Yes	No	Yes	Yes	No	Yes	Yes	No	No	Yes	No	No	No	Yes	Yes	No	No	Yes	Low
Al Shehri et al., 2018	Yes	Yes	No	No	Yes	No	Yes	Yes	No	No	Yes	No	No	No	Yes	Yes	No	No	No	Low
Al Shehri et al., 2021	Yes	Yes	No	No	Yes	No	Yes	Yes	Yes	No	Yes	No	No	Yes	Yes	Yes	Yes	Yes	Yes	Moderate

## Data Availability

Study data are available from the corresponding author on request.

## Supplementary Materials

The following supporting information can be downloaded at: https://www.mdpi.com/article/10.3390/ijerph192416633/s1, Supplementary File S1: MeSH words. PRISMA checklist. Table S1. Excluded studies.
