# Supplementary material for: Oral Health Status, Oral Health Behaviors, and Oral Health Care Utilization among Persons with Disabilities in Saudi Arabia
Source: Int J Environ Res Public Health. 2022 Dec 11;19(24):16633. doi: 10.3390/ijerph192416633 (PMC9778877; doi:10.3390/ijerph192416633)
Supplement: Supplementary file 1 [file ijerph-19-16633-s001.zip › Supplementary File S1-MeSH words.pdf]

MeSH/keywords *(PubMED/Medline, Embase, ISI Web of Science, Scopus, ClinicalTrials.gov and CEN-TRAL):*

[((dental health) OR (oral healthcare) OR (oral health) OR (dental caries) OR (periodontal health) OR (periodontitis) OR (oral health status) OR (oral hygiene) OR (oral health measures) OR (cariogenic diet) OR (toothbrushing) OR (smoking) OR (oral health barrier) OR (oral health accessibility) OR ((oral health barrier) OR (barriers to oral health)) OR ((oral health accessibility) OR (access to oral health)) AND ((Saudi Arabia) OR (Kingdom of Saudi Arabia)) AND ((cerebral palsy) OR (autism) OR (Asperger syndrome) OR (cerebral palsy) OR (Down syndrome) OR (dyslexia) OR (dyscalculia) OR (dyspraxia) OR (dysgraphia) OR (blindness) OR (visual impairment) OR (deafness) OR (hearing loss) OR (hearing impairment) OR (Attention deficit hyperactivity disorder) OR (cystic fibrosis) OR (learning disability) OR (developmental disorder) OR (learning disorder) OR (intellectual disability) OR (autism) OR (autism spectrum) OR (hearing impaired))].

Google/Google Scholar: Search results limited to 20 pages. Above MeSH terms were used as keywords.
