# Supplementary material for: Oral Health Status, Oral Health Behaviors, and Oral Health Care Utilization among Persons with Disabilities in Saudi Arabia
Source: Int J Environ Res Public Health. 2022 Dec 11;19(24):16633. doi: 10.3390/ijerph192416633 (PMC9778877; doi:10.3390/ijerph192416633)
Supplement: Supplementary file 1 [file ijerph-19-16633-s001.zip › Table S1 - Excluded Studies.pdf]

Study	Reason for exclusion
Shenkin, Jonathan D., Martin J. Davis, and Stephen B. Corbin. "The oral health of special needs children: dentistry's challenge to provide care." *Journal of Dentistry for Children* 68.3 (2001): 201-20	Non-Saudi population studied
Davidson, Natasha, et al. "Holes a plenty: oral health status a major issue for newly arrived refugees in Australia." *Australian dental journal* 51.4 (2006): 306-311.	Non-Saudi population studied
Alkahtani, Zuhair M., et al. "Saudi and US dental student attitudes toward treating individuals with developmental disabilities." *Journal of Dental Education* 78.8 (2014): 1145-1153.	Patients not studied
Vellappally, Sajith, et al. "The prevalence of malocclusion and its association with dental caries among 12-18-year-old disabled adolescents." *BMC oral health* 14.1 (2014): 1-7.	Non-Saudi population studied
AlHumaid, Jehan, et al. "Effectiveness of the D-TERMINED program of repetitive tasking for children with autism spectrum disorder." *Journal of Dentistry for Children* 83.1 (2016): 16-21	Non-Saudi population studied
Sedky, Nabila A. "Assessment of oral and dental health status in children with cerebral palsy: An exploratory study." *International journal of health sciences* 12.1 (2018): 4.	Non-Saudi population studied
Alumran, Arwa, et al. "Are dental care providers in Saudi Arabia prepared to treat patients with special needs?." *Journal of Multidisciplinary Healthcare* 12 (2019): 281.	Patients not studied
Alshatrat, Sabha Mahmoud, Isra Abdelkarim Al-Bakri, and Wael Mousa Al-Omari. "Dental service utilization and barriers to dental care for individuals with autism spectrum disorder in Jordan: A case-control study." *International Journal of Dentistry* 2020 (2020).	Non-Saudi population studied
Barry, Mohammed J., et al. "A comparison of salivary mercury levels in children with attention deficit/hyperactivity disorder when compared to age-matched controls: a case-control observational study." *The Journal of Contemporary Dental Practice* 21.2 (2020): 129-132.	Oral health not studied
Abdulhaq, Ahmed, et al. "Tongue microbiome in children with autism spectrum disorder." *Journal of oral microbiology* 13.1 (2021): 1936434.	Microbial study only
Al Daajani, Manal Matar, et al. "Prevalence of Health Problems Targeted by the National School-Based Screening Program among Primary School Students in Saudi Arabia, 2019." *Healthcare*. Vol. 9. No. 10. MDPI, 2021.	Inappropriate methodology
Alkhateeb, Alaa A., et al. "Untreated dental disease and lung transplant waitlist evaluation time for individuals with cystic fibrosis." *Special Care in Dentistry* 41.4 (2021): 489-497.	Non-Saudi population studied

Alshatrat, Sabha Mahmoud, et al. "Oral health knowledge and dental behavior among individuals with autism in Jordan: a case–control study." *BMC Oral Health* 21.1 (2021): 1-8.	Non-Saudi population studied
Moin, Maria, et al. "Impact of oral health educational interventions on oral hygiene status of children with hearing loss: A randomized controlled trial." *BioMed research international* 2021 (2021).	Non-Saudi population studied
Aloufi, Abdullah, et al. "Factors determining the need for general anesthesia to deliver dental treatment for adults with intellectual and developmental disabilities." *Saudi Journal of Anaesthesia* 16.1 (2022): 24.	Oral health not studied
